# On the Analogies and Differences between Typhus and Typhoid Fever

**Published:** 1839-10

**Authors:** 


### Art. IX.

1. *Deux Mémoires, en réponse à cette question—" Faire connoitre les Analogies et les Differences qui existent entre le Typhus et la Fièvre Typhoide."* Par MM. C. E. S. Gaultier de Claubry et J. J. H. Montault. Mémoires de l'Académie de Médecine. Vol. VII. 1838.

*On the Analogies and Differences between Typhus and Typhoid Fever.* By MM. Gaultier de Claubry and Montault.

2. *A Report founded on the cases of Typhoid Fever, or the common continued Fever of New England, which occurred in the Massachusetts General Hospital, from the opening of that Institution, in September,* 1821, *to the end of* 1835. By James Jackson, m.d.—*Boston,* 1838. 8vo, pp. 95.

3. *A Short Treatise on Typhus Fever.* By G. L. Roupell, m.d.—*London,* 1839. 8vo, pp. 301.

4. *Etudes Cliniques sur divers points de l'Histoire des Fièvres Bilieuses et Typhoides.* Par le Docteur H. C. Lombard.—*Genève,* 1838. 8vo, pp. 40.

*Clinical Remarks on certain points in the History of Bilious and Typhoid Fevers.* By Dr. Lombard, of Geneva.

5. *Der Typhus und dessen Erscheinungen, oder die Typhoseptosen, pathogenetisch und therapeutisch erläutert.* Von L. Buzorini, m.d. &c. &c.—*Stuttgart,* 1836. 8vo, pp. 303.

*Typhus and its Phenomena, or the Typhoseptoses; pathologically and therapeutically illustrated.* By L. Buzorini, m.d.

6. *Das Blutfieber, vorzuglich in seiner Verbindung mit einigen Krankheiten des Darmkanals; pathogenetisch und therapeutisch erläutert.* Von F. Kehrer, m.d. &c.—*Mainz,* 1837. 8vo, pp. 128.

*Blood-Fever, especially in its Connexion with certain Diseases of the Alimentary Canal; pathologically and therapeutically illustrated.* By F. Kehrer, m.d. &c.

The seventh volume of the Mémoires de l'Académie de Médecine is almost entirely occupied by two prize essays on the subject of Fever. By the majority of the profession in this country "the analogies and differences" between typhus and typhoid fever, will be regarded but as variations of form and manifestations of the same disease. There is a defect in the mode of putting the question as proposed by the Academy, which considerably prejudices it. It assumes, as it were, that there are two diseases, actually as well as nominally distinguished, whereas this distinction is the point at issue. The two French authors take opposite views of the subject: M. de Claubry contending that the terms typhus and typhoid fever include one disease, M. Montault taking an opposite view, and regarding the terms as properly applicable to two diseases. There is a question which necessarily suggests itself to enquiries of the kind before us, i. e. "what is to be considered as constituting distinction of diseases?" And it is likewise necessary to be well guarded against the influence of names in studying the complicated phenomena of disease. We can easily conceive that if it were proposed to show that under the term variola, two or more diseases were comprehended, it would be no difficult matter to make out a strong case for the affirmative of the proposition, by an ingenious classification of causes, of varying symptoms, of different degrees of morbid changes, occurring during life and detected after death; and in our view of the subject, this is what has by some been effected for typhus fever. With the influence produced by the trammels of nomenclature and ineffectual attempts at definition where definition is almost useless, without a due regard to the analogy of disease, without a comprehensive view of the particular series of morbid phenomena under consideration, as influenced by climate and season, by national or individual characteristics, certain phenomena have been selected as justifying distinctions which, we think, are unfounded in nature. But it is not without feeling the great difficulties attendant on the subject, nor without an acknowledgment of the respect which is due to many whose views are opposed to our own, that we thus state our opinions. It must be admitted that one cause (the contagion of fever, for instance,) acting on two different organizations will not produce similar results; and if the external cause or causes be varied and modified in their proportions and combinations and facilities of action, and the organizations on which they operate are susceptible of their influence in all possible varieties, differences of symptoms and of morbid changes will exist in all possible degrees, the diseases still being identical. There may be extremes of these differences, at one of which certain phenomena are more constantly present, at the other of which they are rarely found to exist, and in the intermediate links the phenomena are associated in all conceivable degrees. Such, we think, are the views that our present knowledge justifies us in taking of typhus fever. We shall make no further allusion (as no attempt in the volume before us is made to prove it) to the following bold and truly characteristic generalization. "I have shown," says M. Lombard, "that there exist in England two kinds of continued fever, well distinguished the one from the other: a sporadic fever, but little contagious, existing in all parts of England, and which does not differ from our typhoid fever; and an exceedingly contagious fever, which commences and has its nucleus in Ireland, whence it extends to England and Scotland, following with great exactness the migrations of the labouring Irish."

It is not our intention to follow the French authors through their most elaborate compositions. It was necessary for them to write what, happily, we may very greatly condense without damage to the question treated of. We shall collect from their papers, and comment upon the chief points which have been regarded as constituting a distinction between typhus and typhoid fever; the minor matters being such as, even if very precise information could be gained respecting them, are not worth the trouble of noticing. Having briefly alluded to the works of the French authors, we shall consider those which follow in the order above given.

The chief points which have been regarded as distinguishing typhus from typhoid fever are mentioned in Dr. Marshall Hall's late lectures. He says, "it seems probable that the *real typhus* is distinguished from *typhoid* fever by being contagious, by attacking old as well as young subjects, and by not presenting the phenomena of disease of Peyer's and Brunner's glands of the intestines." If to this we add the rosy exanthematous spots which by some are also mentioned as belonging to typhoid fever, we shall have selected all the important points on which to rest the decision of this question. If, then, it can be shown that the disease termed typhoid fever is contagious, that all ages are affected alike by it and by typhus, and if neither on the disease of the intestines nor on the existence of any particular exanthema any distinction can be maintained, the inference must be that it is incorrect to speak of two diseases, distinguished as typhus and typhoid fever.

The general evidence on contagion is one on which we have neither room nor is it necessary to enter. There seems no doubt that in the particular case before us, the same kind of evidence can be adduced in favour of the contagiousness of what is termed typhoid fever as of typhus. Our readers will not think the following facts, quoted from the essay of M. de Claubry, unimportant:

"A man and his wife, aged each thirty years, and their two children, aged seven and nine years, were attacked with a *typhoid fever* in this village. A young woman, seventeen years of age, their servant, took the disease, and went away to her own village to be cured. This place was considerably distant from the former, and between them was no direct communication. The mother of the young woman, aged forty, and her sister, aged twenty-two, and a child, aged five years, caught the same disease whilst attending to the young woman. . . . . An epidemic broke out among the scholars of the school of Saumur, a town where typhoid fever had existed some time. Of twenty-eight scholars, who left school for the holidays and went home, and who then became very ill, eight communicated the disease; among other instances, one of these transmitted the disease to her sister, the latter to her servant, and the servant to a friend who came to see her. . . . A servant left his master to visit a relative, affected with typhoid fever, who was living at a distance of two leagues. Shortly after his return he was attacked with the same disease. A person who was nursing a child in the house visited the patient and caught the contagion. At the same time, a woman of the village, who washed the linen of the latter individual, but who had had no direct communication with either of the sufferers, complained of the exceedingly disagreeable smell of the linen which she had washed, was affected with rigors, headach, and eventually with all the phenomena of typhoid fever." (p. 120.)

In the work before us there are many other instances of this kind. They have been explained on other grounds than that of contagion; but as it regards the argument respecting the identity or non-identity of typhus or typhoid fever this is insignificant; it suffices for us that the evidence in either case is precisely of the same character, and if accepted for one must be accepted for the other. Now, having these facts before us, we may (nay, we must,) neglect any inferences which are drawn from what are called *negative facts*. It is a waste of time to consider them, and we shall therefore pass them over. But we cannot omit to quote, from M. Lombard's essay, some remarks bearing on this subject, wherein he speaks of typhoid fever. Whilst enumerating the reasons which induce him to believe in the contagiousness of this fever, he says:

"First of all, during the four years that I have acted as physician to the civil and military hospital of Geneva, two of the male attendants on the patients have been attacked with typhoid fever. Secondly, numerous instances have occurred of the transmission of the disease to different members of the same family or to different inhabitants of the same house. Thirdly, I have followed the propagation of the disease among fifteen individuals successively affected by it. The particulars of the above fact are as follows: During the autumn of 1835, I attended the daughter of a carpenter who lived in the suburbs of the town. She was suffering from typhoid fever, together with one of her aunts who had nursed her during the early period of her disease. As their room was very small they were moved to another room adjoining a corridor. One of the two speedily became convalescent and frequented this corridor, which belonged also to another family. In a few days, a little girl, aged twelve years, belonging to the latter family, and who was frequently in the corridor, was attacked with typhoid fever. A servant who attended on her was likewise affected with the fever, which then attacked another servant who had succeeded to the duties of the former. The sister of the last patient, who spent two nights with her, next had the fever. She was admitted to the hospital and died of the disease. A priest, who had visited the little girl previously mentioned, caught the disease and died, and from him it passed to a servant who attended him, and who likewise died. A female who had visited the girl previously alluded to, and who was circumstanced very differently, both as it regarded fortune and habitation, from the latter, was attacked with the same disease, which lasted seven or eight weeks. An English lady who lived in the same house with the last person, and was frequently in her company, had the fever, which also attacked two daughters of this lady in succession. Two other ladies living in the same house had the fever and recovered, but another was affected by it and died." (p. 17.)

In considering the question of the *age* most liable to be affected by either what is termed typhoid fever or typhus, we must bear in mind that certain ages are liable more than others to certain diseases; but that when such diseases occur at unusual periods of life, they are not on that account regarded as different diseases. On this subject M. Montault observes:

"Youth appears favorable to the development of typhoid fever; but M. Bretonneau has seen the disease in a child of four years, and it is not rare in the Parisian hospitals to see the disease at the ages of four, six, eight, and ten years. And, on the other hand, MM. Petit and Serres have seen an old man die of typhoid fever aged sixty, Andral has witnessed it after seventy years. It is, however, particularly between the ages of twenty and thirty that the typhoid fevers have been most frequently observed. Thus of 88 cases, Louis records 54 between the ages of twenty and thirty. Chomel states, that of 117 there were 91 between the ages of eighteen and thirty, and of 74 cases observed by the author, 43 were between twenty and thirty years old." (p. 190.)

The above quotation is decisive on the question of distinction from age.

We think that the condition of Peyer's and Brunner's glands can, as little as the previous circumstances of contagiousness and period of life, be referred to as any ground for the distinction of typhus and typhoid fever. We know well, in this country, that individuals lie in the same fever wards, at the same period, having contracted a disease to all external appearance similar, with the same or nearly identical symptoms; and that, after death, in some of these cases there may be traces of intestinal and mesenteric disease, in others none at all: and this difference has fairly admitted of explanation, on the principles already alluded to, without having recourse to the theory of distinct diseases. To obtain thoroughly satisfactory *evidence* on this head requires a very extensive geographical consideration of fevers; and we believe that such a consideration would tend to destroy both the theory of symptomatic fever, and of the diversity of what is called two diseases, the fever under consideration. On this part of our subject we shall make some extracts from the essay of M. de Claubry, conveying, as they do, our own views on the subject.

In *typhus*, the inflammation of the surface of the intestines, the rosy colour of the peritoneal membrane of the small intestines in many parts, the deep redness of the same membrane, have been remarked by Pringle, Delbosq, Gilbert, Pellerin. Livid, brownish, violet, and almost black spots have been found upon the peritoneal surface of the small intestine by Fauverges, Reveillé-Parise, Thouvenel, Ducastaing, and Pellerin; and these last observers, in those parts of the internal membrane corresponding to these spots, and chiefly towards the extremity of the small intestine in the cæcum, have found erosions with elevated borders, in the centre of which the peritoneal coat was exposed by the destruction of the other intestinal coats. Others, less exact in the use of terms, and less exact in their descriptions of alterations which they have observed, speak of having found the mucous membrane inflamed, gangrenous in certain points; but M. Tort states positively that he found the small intestines scattered over with gangrenous scars. There can be no doubt that in these instances a similar change in appearance, situation, and pathological character is spoken of. Ducastaing and Pellerin speaks also of swelling, softening, and the reddish-gray appearance of the mesenteric glands, in those parts of the mesentery corresponding to the intestinal ulcerations." (p. 80.)

After having described the changes said to characterize the *typhoid fever*, and which are taken from the well-known works of Louis and Chomel, M. de Claubry continues, "if now we compare these results, are we not necessarily led to the conclusion, that in the *typhus* of camps and the *typhoid fever*, there is not merely a greater or less analogy but an incontestible identity of organic lesions?" We do not know that any additional facts from the morbid anatomy of the intestinal canal are required. The author, who takes up the opposite view to M. de Claubry, omits the evidence just alluded to, and consequently his remarks are valueless. In our own country we do not require any arguments to persuade us of the identity of these supposed distinct diseases; the evidence is constantly before our eyes. The last circumstance to which we shall allude is the occurrence of *a rash*, which has by some been regarded as distinctive of typhoid fever. On this head it is remarked by M. de Claubry:

"This rash is constant in *typhus*. If it has not been always described, because not observed, it may be said, as an excuse, that the same eruption, so constant also in *typhoid fever*, is not once mentioned in upwards of forty cases of this disease collected by M. Petit; his attention, as well as that of M. Serres, having been directed to another series of phenomena. And it is important to observe that this rosy exanthema of typhus, sometimes limited to a small number of distinct spots upon the abdomen, exists also and in greater abundance on the chest, back, and limbs. Eighteen of the twenty-four observers whose works we have quoted speak expressly of this eruption; four speak of it as occurring after the fourth day; two after the fifth; eight in the course of the sixth or seventh; all speak of its appearance between the seventh and tenth day." (p. 75.)

We shall make no further remarks on the works of the French authors, the facts and arguments which we have adduced being such as to satisfy our own minds that other than the reasons generally given must be brought forward to prove that typhus and typhoid fever are terms properly applicable to different diseases. And on this point it is satisfactory to find that Dr. Roupell's opinion coincides with our own. He says "the impression on my mind, arising from the correspondence between the leading features as already detailed, is that the two fevers are identical."

We shall here shortly allude to some remarks of Lombard, if it be but to show the difficulty which attends the attempt to subdivide, on any satisfactory grounds, various forms of fever; and by applying to these remarks the observations which we have already made, we believe that the most satisfactory solution of the differences is obtained.

"In arriving at the conclusions that two distinct diseases exist, a bilious fever and a typhoid fever, I have been brought to recognize that in mild cases it is exceedingly difficult to distinguish them, and that a great number of cases, commonly designated gastric disturbance (embarras gastrique) or bilious fever, are in fact cases of typhoid fever, presenting the majority of symptoms characteristic of this affection. And moreover, in one case which terminated in violent death, I found all the characteristic lesions of typhoid fever in an individual who had worked up to the moment of his death." (p. 38.)

The questions which it is especially the object of M. Lombard to answer, are: Is there such a disease as a bilious fever independently of any gastro-hepatic inflammation, and different from typhoid fever? And are there any well-marked characters by which to distinguish a bilious from a typhoid fever, or are these fevers only different degrees of the dothinenteritis? The cases, falling under the observation of any single individual are very few by which this question can be decided. But M. Lombard has met with some, having an important bearing on the question. We shall here give an epitome of his remarks.

"A simple gastric disturbance, febrile or non-febrile, is not difficult to distinguish from well characterized typhoid fever; but if the latter is mild, and the characteristic symptoms are partly absent, the distinction becomes more difficult, and if a gastric disturbance is prolonged, is accompanied by nervous symptoms, by diarrhœa, and typhoid eruption, it is difficult to distinguish this from typhoid fever. Does pathological anatomy afford any means of distinction? Bilious fever in these diseases rarely terminates in death; but cases where the series of symptoms must be regarded as characteristic of bilious fever, have terminated fatally. It is difficult to determine absolutely the absence of gastro-hepatic inflammation in the cases called bilious fever; but if it is remembered that diuretics and purgatives must necessarily aggravate the disease, if it depended on such inflammation, one is compelled to admit that cases do exist in which the bilious element of the disease constitutes a more important part than the inflammatory. Besides, the progress of bilious fevers, which frequently last two or three weeks, without marked aggravation or diminution, may be regarded as constituting a marked difference between them and inflammatory diseases. And in the cases which have terminated in death there is occasionally found no trace of hepatic or gastro-intestinal inflammation, so that one is compelled to admit the existence of a bilious disease differing from hepatitis or from gastro-enteritis." (pp. 4,5.)

Two cases of bilious fever are here mentioned by the author. The latter ended in death, and as it is from the examination after death more complete than the former, we shall quote it entire.

"A female, æt. fifty-eight, sent for me after having been ill fifteen days; she was then suffering from abundant bilious vomitings, frequent yellow stools, great prostration, dry, red tongue, slight headach, but no vertigo, no tinnitus aurium, no deafness; the abdomen was somewhat tender on pressure of the epigastric region, the pulse rather slow, the skin hot and burning; there was no trace of typhoid eruption. The vomiting and diarrhœa resisted all the medicines employed, and there was not a typhoid symptom during the six weeks that the disease lasted. After death, no appearance could be found by which to account for death. The mucous membrane of the stomach was neither thickened, nor injected, nor softened, but of completely normal appearance. Neither the gland of Peyer nor the agminated glands were apparent; there was neither ulceration nor recent cicatrix around the valve or in the interior part of the ileum. The large intestine was likewise free from morbid change; the liver was normal, the gall-bladder distended by liquid green bile." (p. 5.)

On these cases the author remarks that in the first, the age of the patient (seventy-four years) and the absence of every symptom of disease of the nervous centres would not allow the disease to be regarded as typhoid fever; and in the second case there could be no room for doubt, for on examination after death, no disease of the glands of Peyer was detected. The second case could not be referred to an inflammation of the liver, stomach, or duodenum, because in none of these organs was there any appreciable change attributable to inflammation; and if it is remembered that the disease continued six or seven weeks, ending in death, the anatomical appearances should have been such as to leave no doubt of their inflammatory origin; but nothing was found but a superabundance of bile in the intestinal canal: whence it must be inferred that the "polycholie" or bilious disease actually does exist, and ought to be distinguished both from typhoid fever and from hepatic or gastro-duodenal inflammation.

Having decided the first question by morbid anatomy, we come to the second: Are there symptoms of bilious fevers distinct from those of typhoid fevers? The cases related show that there are instances of bilious disease, the symptoms of which have no resemblance with those of typhoid fever. These are well-marked cases; the distinction consisting in the absence of typhoid symptoms, the continuance of vomiting and diarrhœa, and the absence of the lenticular eruption upon the abdomen. But it is far from being the case that all bilious fevers are thus marked and distinct, and in certain circumstances the reunion of the different symptoms renders the diagnosis somewhat difficult. M. Lombard says he has verified the observation of Louis, that there exist cases of typhoid fever of so light a character, that individuals so affected are not confined to their beds, but continue their usual occupations; and from all which he knows of the subject, he concludes that there are insensible degrees between the most slight cases of gastric disturbance and the most serious typhoid fever. Cases illustrative of this opinion are given, in which the gradations are exemplified, and concerning which it is remarked that it is difficult to establish between the two orders of disease any other distinction than one of degree. On this subject, and after having quoted other cases illustrative of the above views, M. Lombard concludes:

"The facts collected justify the inference that there are insensible degrees between a simple "embarras gastrique" and the most severe typhoid fever; but it does not thence follow that there are no true bilious diseases and no true gastric derangements, because we have cited cases of this kind which have terminated by death without presenting any of the lesions characteristic of typhoid fever; only it appears very difficult to distinguish if a mild case of gastric derangement arises from a simple derangement of the alimentary canal, or if, as in a case related of a suicide, it is accompanied by a development of the glands of Peyer. Perhaps in the lenticular eruption may be found the distinctive sign of the intestinal eruption and of the bilious disease. But further observations are necessary to determine this in at all a satisfactory manner." (p. 16.)

Of the truth of this last remark we are fully convinced, and we are therefore only disposed to ask other questions in reply to the questions and statements of M. Lombard. How is typhoid fever to be defined? Is it the same as typhus? What number of exceptions is to be admitted in applying the definition? Is it necessary that there should be any absolutely uniform morbid appearances after death? In what proportion of cases is the rash to be looked for, so as to justify any precise inference from its absence alone, or from its absence together with some other symptom of equally frequent occurrence? May it not happen (supposing the disease, typhus or typhoid, to be produced by a poison,) that in the fatal case quoted above, the poison may have expended itself upon one organ in particular, "the bilious element," as it is termed, being the predominant element in the disease? But in this case, it must be remembered that, although it is said that there were no affections of the nervous centres, there was "great prostration" and "some headach." What are the cases termed by the author "bilious sub-typhoid" fever? Are they not examples of the combination of effects of the same causes? Much that we have said in a previous part of this paper may be applied in reply to the above queries and in answer to M. Lombard. But we consider that the question of a bilious fever as distinct from a typhoid fever is one concerning which we require much further information than we now possess, to enable us to decide.

The very excellent "Report on Typhoid Fever," written by Dr. Jackson, is founded on 303 cases, considered according to the numerical method. It must, as is stated, have cost its author "an amount of labour to arrive at its details, which could hardly be credited by one who never engaged in a similar work." Dr. Jackson entertains the views of Louis respecting typhoid fever, and believes in its being distinct from other forms of idiopathic fever. He says "it is plain that there are at least two species of continued fever, both in Europe and in this country; and further researches may very possibly show more." But there are no sufficient reasons given in Dr. Jackson's work for maintaining the distinction. The point mainly relied on by him is the affection of the intestinal glands and mucous membrane. We have already considered the weight which should be allowed to this condition in deciding on differences between typhus and typhoid fevers. In the course of our remarks we shall have other opportunities of referring to Dr. Jackson's work, which, however, does not contain much matter of interest to the reader. We should, however, not omit to mention that the usual affection of the glands of Peyer is mentioned as having been found in all the fatal cases of typhus mentioned by Dr. Jackson.

In the preface to Dr. Roupell's work, it is stated that its objects are "to assert the claim of the prevailing epidemic to be ranked among specific fevers, to separate it from some with which it has long been improperly confounded, to show at the same time its analogy with others, and to improve the pathology of all." Dr. Roupell remarks that, "it is owing to a certain specific cause. For, when closely observed, it has been found to pursue a certain definite course, passing through its stages with regularity, spreading by infection, and being marked in its progress by a distinctive rash. Here then we have all the characteristics of the genuine exanthemata of authors, to which class it seems correctly and exclusively to belong."

"Improving the pathology of all fevers" is an object well worthy of the efforts of any physician; for, much as we do know of them at present, there remains very much with which we somewhat vainly seek to become acquainted.

There is much of Dr. Roupell's book confirmatory of an opinion, not originally but somewhat decisively, stated by Dr. Peebles, in the forty-fourth volume of the Edinburgh Medical and Surgical Journal, and which we are surprised that Dr. Roupell did not notice. Dr. Peebles writes as follows:

"I have endeavoured to trace the history of the petechial contagious fever which has been the subject of our enquiry, and I trust that I have shown that such a disease existed from the earliest ages, that the fever is of a peculiar type similar to that of exanthematous diseases, and particularly that it is accompanied with an eruption as distinct and specific as any other *exanthema*, and which should be considered as pathognomonic of the disease; that it is produced by a miasm or poison *sui generis*, and is capable of propagating itself like other contagious diseases. In bringing our observations to bear on the contagious fever of this country, we shall find that the leading characters of the petechial disease and the sources from which it flows are very much the same with those of our typhus fever. The sthenic or irritative sthenic type of the fever early in the disease, the tendency to the asthenic and putrid state towards the latter stages, the sudden and great prostration of strength, the condition of the tongue and eyes and the expression of countenance, the constant invasion of the cerebral and pulmonary system, and particularly the exanthematous eruption which always accompanies the fever, are characters common to both. This eruption has been already noticed occasionally by some practitioners in this country when the fever became epidemic; but, as I have stated, I have found it as constant as any exanthema of other eruptive diseases, and during two years of attentive enquiry I have found the contagious fever of this country to correspond very closely in the above characters, with the difference that the exanthematous affection is less profuse in a majority of cases. This may arise from the disease not being epidemic at present, for it is then much more distinct and copious. But the character of the eruption and the period of its appearance are still precisely the same. Dr. Lorimer, Physician's Clerk at the Royal Infirmary, who has assisted me for a year and a half in examining fever patients on their admission, informs me that he has observed the exanthema in every case, with a few exceptions, when the patient was brought into the hospital before the eighth day of the fever. (p. 373.)

All the remarks of Dr. Roupell are confirmatory of the above view. And that in certain localities (in the two instances just alluded to, in large and crowded cities,) the fever termed typhus may be marked by the chief characteristics of an exanthematous fever, must be admitted. It remains to be seen whether, in other parts of the country, the same phenomena characterize the disease; and, as the general attention has been more called to the subject, it is to be hoped that the condition of the skin and the series of symptoms occurring in the disease will be more accurately noticed and defined than has hitherto, too frequently, been the case. Dr. Roupell states that in his note-book "the rash is recorded in 70 out of 100 cases promiscuously taken, no mention being made of it in the remaining 30; again, out of 100, all of whom had the rash, it was present in 86 on their admission, and showed itself subsequently in the rest." We quote the following from Dr. Jackson's Report as valuable evidence, especially because he has no theory to support.

"Rose spots were not noticed until the year 1833. In that and the two following years they were noticed in 70 cases; in some of these, however, they were very few in number. Sometimes only three or four were seen; but in a few cases they were numerous, covering the trunk, and appearing even on the limbs. In the years 1833-35 inclusive, there were 106 cases, so that ($106 \div 70 = 1{\cdot}51$), 1 in 1·51, or about 2 in 3 had rose spots. I am not at all satisfied, however, that they did not escape notice in some cases."

The following is the description given by Dr. Roupell of the rash, which must be carefully distinguished from petechial spots.

"It is of a red colour, the shades of which are various: in some cases bright and vivid, more generally, however, dusky and undertoned; if the rash is fully developed, the cuticle is slightly elevated; and when the vessels are very turgid, the eruption is sensible to the touch. It is most commonly found on the chest, trunk, and limbs; sometimes on the face, and was noticed in a recent case to have reached and occupied the scalp; nor is it confined to the outer surface of the body, but extends itself to the lips and lining membrane of the mouth. .... It appears in spots or patches, circular in form, and varying in size from that of a pin's head to that of a pea. No itching attends its presence on the skin, nor is desquamation of the cuticle an ordinary consequence. .... The duration of the rash in typhus, according to M. Chomel, is from three to four days; should any mottling of the skin appear after this period, he attributes it to a fresh eruption. It may here be observed, that in some cases, when the rash could not be perceived, but in which it was deemed advisable to bleed the patient, and a ligature was applied for that purpose to the arm, the eruption appeared below the tape. Acting upon this idea, I have at other times been able to exhibit it by artificial congestion of the vessels." (pp. 35-37.)

That the rash should be occasionally or even frequently absent need not surprise those who are conversant with exanthematous fevers; especially if they are considered in their analogies with other diseased actions produced by poisons upon the organism. The frequent occurrence of sore-throat, during the prevalence of scarlatina, with the scarlet injection of the mucous membrane of the fauces is, amongst others of a similar kind, a fact of great importance in reasoning on the nature of exanthematous fevers; and a little reflection will convince us that the condition of the skin in these diseases may have had more attention paid to it than equally uniform changes in other parts.

We are far from determining on the universal occurrence in typhus fever of those peculiarities mentioned by Dr. Roupell. Our disposition is to believe that the disease described by Dr. Roupell, Dr. Peebles, and others, is essentially the same as that which is generally denominated typhus, modified according to circumstances to which we have already alluded; but it is an inference which cannot, at present, rest on any satisfactory grounds. Before determining the question as to the propriety of classing typhus among exanthematous fevers, we must obtain far more extensive information than we already possess. From what we do possess we are disposed to admit that, with probably greater irregularity as to the course of the disease, less protection against its recurrence, a greater variety of morbid phenomena, and probably a more extensive sphere of action, typhus is closely allied to exanthematous fevers. Dr. Roupell's experience is amply confirmatory of the infectious character of the disease. Numerous cases are related of typhus with hemorrhage from various organs, with erysipelas, with purulent deposits in the cellular membrane, with carbuncle, with extensive sloughing, with sloughing phagedæna, and other morbid changes, both organic and functional. Dr. Roupell is disposed to account for many of the symptoms of typhus in its worse forms by supposing that pus is mixed and circulates with the blood.

"We see that inflammation of a vein and admixture of morbid secretions from the inflamed part with the blood causes, on ordinary occasions, typhoid symptoms, and gives rise to a fever, attended by subsultus tendinum, low muttering delirium, and a black tongue. The similarity of the symptoms in phlebitis and in one stage or period of typhus gives reasonable ground for belief that the corresponding appearances in the two diseases arise from a not dissimilar cause, and hence we see an explanation of one great train of effects in typhus, and learn how typhoid symptoms, appearing in various diseases, both chronic and acute, may easily and naturally be confounded with real typhus. Hence we see how readily what is called spontaneous typhus can be generated." (p. 124.)

This quotation, which affords another instance of the necessity, now more generally felt, of conceding to the fluids a large share in the production of various diseased states, receives further confirmation from the observations of Dr. Ferguson, recorded in the last Number of this Journal. We particularly allude to his remarks on that which he terms the complicated form of puerperal fever. Dr. Roupell has taken great pains to establish the fact of an inflammation of the minuter vessels, and the results of such inflammation, as the cause of the phenomena above alluded to. We recommend to the perusal of our readers the sections wherein, both from facts directly bearing on the subject and arguments from analogy, he endeavours to establish his theory. Indeed, throughout Dr. Roupell's volume there is much ingenious speculation, the result of much thought on the intricacies of fever, and which are well deserving of attention. But we must confess that—seeing the many objections which may be brought forward against all existing theories of fever, and, as we are about to occupy the attention of our readers with a very ingenious speculation by Dr. Buzorini on the nature of typhoid diseases, which, however, we offer chiefly as an ingenious curiosity,—we are little inclined, at present, to expend more time and space on mere speculation.

There is a useful chapter in Dr. Roupell's work, on the morbid appearances in typhus; and we refer with much satisfaction to some of his remarks on the treatment of the disease. Especially do we regard it as a truth which cannot be too strongly impressed upon the mind that the disease has a specific origin. The following remarks are important:

"The importance of ascertaining the origin of a complaint is of the highest value with respect to treatment. The blood-shot eye, the pain and increased temperature of the head, attended by delirium, offer inducements to depletion, which it is very difficult to resist, and which it would be most unpardonable to overlook, if the disease had not a specific origin. But when once we are satisfied that all the above symptoms arise from the introduction of a poison into the system, we feel that much more important objects are to be obtained than the mere subduing of inflammation, even although we should feel satisfied that such a condition did actually exist. We know that a cause affecting the whole system is now in operation, and that the reduction of action in one part is doing very little, whilst the causes giving rise to it still remain and are capable of producing it elsewhere. A specific disease cannot be cured by depletion; over-excitement can, indeed, be controlled, but the disease still remains in operation." (p. 230.)

We need not allude to the contradictory opinions entertained respecting bleeding in typhus. Dr. Roupell is an advocate for bleeding in the earlier stages of typhus; but this advocacy is modified by the caution which, respecting the same remedy, should be applied to all specific infectious, eruptive diseases. Emetics he considers as valuable.

"The combination usually employed by me is ipecacuanha with antimony; but should the patient be weak and feel such nausea as to induce the belief that emesis will speedily be induced, ipecacuanha may be given alone, its effects being aided by diluent drinks.... The result of this method of treatment is often to relieve the head-ach, induce sleep, provoke perspiration, stop at once all disordered action, or mitigate the formidable indications. Few remedies, in truth, exert such beneficial influence over the disease as emetics, and, with the precaution mentioned above, they are almost universally applicable, and open to no objection whatever." (p. 242.)

Purgatives, carefully employed, are considered by Dr. Roupell as equally valuable with bleeding and emetics. Mercury in ulceration of the bowels is spoken very highly of.

"There is no remedy which can compete with the hydrargyrus c. creta, when ulceration has taken place in the bowels.... The good effects of this remedy in inflammation of mucous membranes is well known, and, when combined with Dover's Powder, as employed at St. Bartholomew's Hospital,* constitutes an excellent formula; given at intervals of four, six, or eight hours, it allays abdominal irritation, checks diarrhœa, and calms general constitutional disturbance." (p. 247.)

The principle laid down by Dr. Roupell for the administration of stimulants is "to maintain strength enough in the heart for the circulation to continue;" to give them only during the existence of real debility. The state of the pulse and coldness of the extremities are the only signs mentioned as indicating the condition when stimulants may properly be administered; but this is not satisfactory as a guide. It is by no means necessary that the extremities should be cold when stimulants are imperiously called for; and the state of the pulse, apart from that of the heart, would give very inadequate assistance in deciding on this most difficult point of practice. Dr. Roupell was, no doubt, unacquainted with the highly important observations of Dr. Stokes on this subject, noticed in our last Number; but we must call the reader's particular attention to them. If, even after Dr. Stokes's remarks, it were true, as Dr. Roupell remarks, that there is no positive sign to go by in this matter, still he might have made the section on stimulants far more useful by mentioning certain conditions, very misleading to the young practitioner and tending greatly to perplex his mind, with which stimulants are not incompatible; and especially should proper cautions have been laid down as to the mode of their administration, and the effects from which we may safely conclude whether or not they should be continued or abandoned. The following remarks on this subject, contained in Dr. Jackson's report, are worthy of attention:

"When a patient is induced to take cordials (vinous liquors) reluctantly, they seldom benefit him, and are often followed by injury. When he is greatly enfeebled, at a late stage of the disease, he may be safely asked if he wishes for them, and if he does, he may try them; they will seldom hurt him then, if he takes no more than is grateful to him. When he spontaneously demands them, as late as the third week, they will almost always be found useful."

On the employment of opium we must quote some observations from Dr. Roupell, which greatly coincide with our own opinions.

"If there is one remedy of which the advantage is undoubted—if the immediate subsidence of untoward symptoms follows the exhibition of any medicine—if future amendment be the test of utility, then is opium a valuable remedy; but it is not to be employed largely at any period, nor resorted to in the first stages, nor when stupor is present or approaching; but if given at that period of the disease when there is

* This consists of equal parts of hydr. c. creta and Dover's Powder, made into a pill with conserve of roses.

increased action without fever, and all the signs which indicate irritation, good will invariably result." (p. 266.)

We should be disposed somewhat to extend the limits here prescribed to the beneficial action of opium, having found great benefit from its use even in the early stage of fever, when an intensity of headach has existed quite out of proportion to the febrile symptoms generally. And we believe that the advantages derivable from the use of opium in the practice of medicine are not at all sufficiently understood; but that certain fears, founded on its supposed physiological action prevent its being employed in many instances where it might be productive of very beneficial effects. On this account we have read with much satisfaction some remarks in the volume lately published by Dr. Holland, which coincide in the main with opinions which we have long entertained. There is nothing of which we are more convinced than that the usual teachings of lecturers and writers on materia medica will, as far as opium is concerned, be found contradicted by actual experiment.

We now come to the consideration of the works of the German authors, which are widely different from those which we have hitherto noticed. It tends to make a reader very tired of theories, when he finds that almost every writer on the subject of fever has his own to support. Dr. Kehrer has attempted to establish a distinct form of fever, which, when existing in its simple state, consists in a condition of irritation of the blood. Dr. Buzorini has devoted a large part of his work to a consideration of the morbid condition of the blood in typhus fever, and in diseases allied to this, and to point out analogies, of which he conceives the existence, between it and blood when deprived of life. He has revived the doctrine of crises, and considers them as essential to the cure of all typhoid diseases: but, as such crises may occur gradually or accompany the whole course of the fever, they are not necessarily very obvious, and may escape any other than the most minute attention. We shall present our readers with a short analysis of his views, with which they cannot fail to be pleased, from their ingenuity, however little they may be satisfied of their truth. It becomes necessary, in fulfilling our office as reviewers of foreign medical literature, to notice occasionally speculations of some ingenuity, but which are open to many objections; and these objections are sometimes so obvious that it is unnecessary to particularize them. There is another reason why we analyze the work of Dr. Buzorini. The electric condition of the animal-body, and its electric relations with all that surrounds it, are subjects of considerable observation with some foreign physicians, as will be seen from our account of M. Goudret's work, in the present Number. Some Germans also believe that, with respect to erysipelas, rheumatism, and other diseases, very opposite electrical states of the body have been found to exist: and, in the history of the causes of disease, the electric condition of the atmosphere forms, among some of them, a very prominent subject of speculation. Many have attributed the sudden appearance and rapid extension of influenza to such a cause, and have endeavoured, with perhaps more ingenuity than success, to associate these with planetary influences and certain mundane phenomena, attended with the development and concentration of electricity. Dr. Buzorini has, both in explaining the development of typhoid diseases and their nature, made considerable use of such electric phenomena. Some, at least, of his observations on this subject will not be unacceptable to our readers. It requires but little reflection to be convinced that our bodies are in a certain relation with electricity, with which we are little, if at all, acquainted. The sensations of oppression which precede a storm attended with lightning and thunder, enable many to predict its approach with certainty, and the sense of lightness and activity which follows such a storm are very generally acknowledged. Few cannot but have noticed the development of electric sparks which takes place when, in certain states of the weather and (we believe) under certain conditions of bodily feeling, we rapidly remove a flannel garment from our bodies. But it is not with electricity in such abundance that Dr. Buzorini and others among his countrymen have to do. The quantities which become the subject of some of their experiments are only appreciable by the electro-magnetic multiplicator; but, by means of this instrument, it is stated by them that they become very perceptible. Without, however, any further preface, we shall briefly notice the works before us.

Dr. Kehrer has endeavoured to establish, under the name of Febris hæmatodes, or blood-fever, a distinct species of this disease. Its characteristic symptoms he states to be *a frequent, large, and soft pulse; general heat of body; abundant evacuations from various organs;* and the negative characteristic, that *no tissue is visibly changed in its structure.* The nature, essence, or proximate cause of this fever is regarded as evinced by the above symptoms, which are considered as indicating a condition of direct irritation of the whole mass of the blood, of which the globules are said to be especially affected; their morbid change consisting in apparent turgidity. The relations borne by this fever to other forms of fever are as follows: *Synocha* (it is said) affects the solids; *nervous fever* affects the nervous substance; *blood-fever* affects the blood. In synocha, there is inflammation of tissues, with its characteristic effects; the local actions call forth the constitutional; there is a considerable manifestation of power, and a coating in the blood. In nervous fever, there are symptoms of disturbance of common sensation, the nervous system being involved. The Febris hæmatodes is opposite in its character to both: it commences by irritation of the general mass, with local congestions of the blood. The simpler the course of this fever, the less do the nerves and other solids participate; but it is sometimes connected with nervous and synochal fever, and in such cases its characteristic symptoms become variously modified. The *largeness* of the pulse is especially alluded to as characterizing this fever, and as depending on the condition of the blood which belongs to it. A sufficiently fanciful explanation is given of this somewhat undefinable characteristic of *largeness.* The parietes of the arteries are supposed to be very extensible and extended; the blood itself to be in a state of turgidity; a condition of independent vital extension, distinct from the state of plethora in which the pulse is said to be *full.* This condition of the blood, it is well observed, does not admit of demonstration, because it ceases with life, or when the blood is drawn from the body.

We have given the above outline of the fever of Dr. Kehrer, which, however, we do not regard as grounded on correct observation. The notion of a simple condition of irritation of the blood, manifesting itself by turgidity of the whole mass, and this blood circulating through the body without affecting the solids, and so constituting a fever which is capable of distinction as blood-fever, is too hypothetical to merit any serious contradiction. We are told, also, that a characteristic of this fever is the absence of any visible structural changes; but that, during its course, the tissues of various organs are implicated,—this implication, however, being only *secondary*,—i. e. *the effect of too violent a critical concentration.* This may or may not be, but the affirmative is not proved; and, as it is neither agreeable nor profitable to stir the question of the origin of fever which is involved in the above, and without a consideration of which (for which we have no room) we could not arrive at any satisfactory conclusion, we shall pass on to the second treatise.

The work of Dr. Buzorini is characterized by very considerable ingenuity, if it is worthy of no higher commendation; and, although its perusal may not throw any more light on the essential nature of fevers, it will afford an example of the mode of speculation in which many German physicians indulge, and of the connexion, of which it is endeavoured to prove the existence, between vital, physical, and chemical processes in the production and phenomena of disease: and it may not be without its advantages in directing attention more to the condition of the blood in fever; the prevalent disposition to regard the solids as the parts chiefly affected therein, either primarily or secondarily, having unquestionably too much drawn away the attention from changes in the blood. We shall therefore present our readers with an analysis of Dr. Buzorini's work, and in as few words as possible.

The word *typhus*, Dr. B. remarks, was applied by Hippocrates, not only to fever with stupor and coma, but to other diseases attended with diminution or loss of sensibility. It is here applied in the same sense; and, by adding *sepsis* (from σηπω, putredinem accerso,) he forms the word *Typhosepsis*, which is intended to designate a family of diseases termed *Typhoseptoses*, and of which typhus (so called) is only one species. *Typhosepsis* is defined as "a morbid process constituted by a tendency to paralysis of the nervous system and of the blood, by the phenomena of reaction of the organism, and by the effects of this combination." Under this definition Buzorini includes various diseases as *local* Typhoseptoses. Typhus fever is regarded as a universal Typhoseptosis; so that it offers all the morbid phenomena and changes which separately constitute the local forms of this diseased process.

There are two constituents of this Typhosepsis, *a lesion of the nervous system* and *a lesion of the blood.* The various known changes in the nervous system are here alluded to, such as injection, change of colour, consistence, &c.; and an altered specific gravity of some parts of the nervous system is said to be one of the most frequent changes observed therein. That some alteration has not always been found is ascribed to want of care in conducting the examination; but it is added, that the most constant sign is change of specific gravity of the brain. The character of the nervous symptoms is feebleness and tendency to paralysis, termed *Neurasthenia typhica.* In addition to the affection of the nervous system, morbid changes in the blood are required to constitute Typhosepsis. The following appearances, in various degrees, are observed in typhoid blood: In the dead body, it is fluid, dissolved, remarkably dark, and little disposed to coagulate. If the disease be much developed, gas is found in the blood, and small vesicles containing air are formed in the veins. The blood smells offensively during life. If death occur during the later stages of typhus, the quantity of blood is greatly diminished in proportion to the size of the body. If blood drawn from a vein be examined, it is seen to be darker than in the healthy state. In the early part of the disease it coagulates quickly, but slowly when the disease is more advanced; and the coagulum is always soft, loose, and easily torn, small in proportion to the serum, and it more readily putrefies than healthy blood. The solid constituents of the blood are diminished. The serum of healthy blood contains, according to Prevost and Dumas, 10·0 albumen, 90·0 water, in 100 parts. In 1000 parts of blood taken from a typhoid patient, Buzorini found from 48 to 60 parts only of albumen. There is likewise a diminution in the quantity of fibrin and colouring matter in the crassamentum. On the other hand, the watery constituents exist in a larger proportion, and the salts are diminished. Buzorini's experiments accord very closely with the following table, published by Dr. Clanny.

| Constituents of Blood. | In the Healthy State. | In Typhus Fever. | | |
|---|---|---|---|---|
| | | First Stage. | Second Stage. | Third Stage. |
| Water . . | 678 | 729 | 772 | 732 |
| Colouring Matter | 160 | 136 | 122 | 130 |
| Albumen . . | 121 | 98 | 75 | 101 |
| Fibrin . . | 28 | 25 | 22 | 26 |
| Salts . . . | 13 | 12 | 9 | 11 |
| | 1·000 | 1·000 | 1·000 | 1·000 |

And he has likewise found, he assures us, that the proportion of solid constituents of the blood increases with the favorable progress of the case. The specific gravity of the blood is lowered with the loss of its solid constituents. When the blood of a fatal case of typhus is examined by the microscope, the globules are seen to have lost their normal form, to have increased in size, and to be without the proper sharpness of their edges. Now, in putrid blood, the globules become rounded, and break or are dissolved; and this circumstance is regarded as pointing out the condition of the blood in typhus: that there is a diminution of the vitality of the blood, shown by its resemblance to blood undergoing decomposition after death. This condition of the blood has been termed increased venosity, scurvy, putridity. It is a tendency, more or less, to *paralysis* of this fluid, a consequence of which is that the blood becomes subjected, in various degrees, to the chemical laws of unorganized bodies. Typhus is regarded as a combination of the above lesions of the nervous system and of the blood. Instead of reaction (such as occurs in simple vascular irritation,) there is a condition of passive vascular irritation, diminishing still more the life of the nervous system of the part. This acts again, by increasing the paralysis of the blood. From this disposition to mutual increase arises the impossibility of a return to a normal condition, until the dead constituents of the blood are thrown off. The changes produced by the congestions in various organs are, if superficially examined, like those of inflammation; but, from the diminished influence of the nervous system and the septic state of the blood a true inflammation is not possible. There is no effort to form new vessels; no organized formations or secretion of pus. The less the typhoid character of the disease is developed, the nearer is the approximation of the phenomena to those of inflammation. As the blood becomes more fluid, organs become softer and more disposed to decomposition,—e. g. softening of mucous membranes, gangrene, putrescency, &c. The more life that remains in the blood and nervous system the less influence has the chemical action, and *vice versâ*. Thus there are two actions constantly going on. The decomposition of blood drawn from a vein is first mechanical,—i. e. coagulation. So also in typhus, when the influence of the nervous system is diminished, the blood separates into fibrin, colouring matter, and serum. The vital power, we have seen, is in proportion to the quantity of fibrin and albumen: so, in typhus, these are diminished; for, as in dead blood, the fibrin and albumen separate, and nature tries to expel them by stool and by membranous secretions in the canal. The fibrin cannot become organized: it is dead. The mucous membranes of the intestines are the chief excretors of albumen in typhus: sometimes this is exuded between its coats, causing ulcers. If the exudation does not take place by the mucous membranes, it occurs by the serous membranes and the cellular tissue. There is an attempt to separate the serum by the skin and serous membranes; and the latter excrete albumen when it does not pass off by the mucous membranes.

The salts of the blood pass off by the kidneys, and also in the excrements; but, in the more developed typhoid cases, the salts do not appear in their original form, but they are separated into acid and base; as, when a stream of electricity is passed through the blood, the acid appearing at one pole, the alkali at the other. In typhus, much free electricity is found on the skin, and the electro-galvanic multiplicator shows this to be galvanic electricity; and hence the reason that the acids appear on the skin at the positive pole, and the alkalies in the intestines at the negative pole; and *vice versâ*. Thus we find acidulous saliva, ammoniacal urine. This is the point of relationship between rheumatism and the Typhoseptoses: in the former, acid secretions (uric, purpuric, rosacic acids,) are frequent; and rheumatic phenomena are not unfrequent at the commencement of typhus. The sudamina, so frequent in typhus, are explicable, together with their acid contents, by the above electric acid. The diminished quantity of salts in the blood admits of a similar explanation. The changes alluded to in the globules of blood occur with the increased quantity of water and the diminished quantity of salts, and evince most remarkably a decomposition similar to that which is undergone by dead blood. The dark sordes on the tongue and gums are owing to excretion of the separated colouring matter. The condition of the blood explains the remarkable prostration in typhus. As the disease advances, all parts of the body become implicated, the bones as well as the softer parts: hence not only are there animal matters in the excrements, but a quantity of phosphate of lime. If the paralysis of the blood is more advanced, gas is developed in it: gas is also sometimes developed beneath the skin, under the form of emphysema; and, in a body dead from typhus fever, an emphysematous condition frequently occurs in a few hours.

The *calor mordax* is the result of an electro-galvanic process, whereby the latent heat in the blood tending to putrefaction, is disengaged. That electricity can develop warmth from blood is shown by the experiments where the temperature of blood drawn from a vein is increased, as soon as the wires of a small galvanic battery are inserted. We know, from the experiments of many physiologists, the influence of electricity on vital phenomena; and it is also known that a constant galvanic process attends vital action; and we likewise know that, in every chemical process, and especially in the transition from an organized to an unorganized state, electricity is the operative power: and thus we observe, in this morbid process, that the organic electricity is changed or increased. The animal organism develops more or less electricity, positive on the skin, negative on the mucous membranes; as has been shown by the electrometer and electro-magnetic multiplicator of Schweigger and Poggendorf. In typhus these electric relations are variously modified. The electrometer of Bohnenberger frequently shows much free negative electricity on the skin; and this is the case where the disease has a tendency to develop itself externally, and when violent sweatings and exanthematous phenomena occur. On the electro-magnetic multiplicator the action of the electricity which is present is very great and very remarkable. We find also that the kind of electricity is connected with the development of the diseased process: thus, when decomposition is great, the electro-galvanic prevails; whilst, in less advanced forms of the disease, the organic electricity shows greater physical power: as in other cases we find that a quantity of electric fluid, capable of communicating a violent shock, possesses very little chemical action, and *vice versâ.* Among the prevailing physical actions, we see exanthemata originate in typhus, and the form is dependent on polarity; whilst among the chief chemical actions are strongly developed putrescencies, and which, according to the electro-galvanic polarity, decide the acid or alkaline nature of an excretion. Thus, for example, the skin, in a normal state, is positively, and the mucous membranes are negatively electric; but in typhus these poles are sometimes reversed, and the ordinarily alkaline secretions of mucous membranes and salivary glands become acid, and the usually acid secretions of the skin alkaline or neutral.

The degree of reaction depends on the energy of the blood and of the nervous system. If the termination be favorable, it is by crisis, by sweating, eruptions, or evacuations from the bowels, &c.; but, as the *vis medicatrix* is constantly acting to restore the proper condition, from the commencement of the disease, the effect is sometimes slowly produced, and crises are not always witnessed.

Atmospheric influences must be regarded as among the most important causes of typhus, and first among these *atmospheric electricity.* Chemical processes, evaporation connected with chemical action, vegetation, &c., are productive of positive electricity. As the vapours ascend, positive electricity ascends with them, and leaves the earth in a negatively electric state. Hence the quantity of positive electricity increases with the height, whilst in the lower strata a portion of the positive electricity is connected with the negative electricity of the earth: but, as the atmosphere is a bad conductor, the whole of it may be regarded as a Leyden phial, the positive extremity being above, the negative below; whilst we live in the isolator, in the intermediate air. Hence the organism is more or less protected from the influence of electricity. Atmospheric electricity, which discharges itself by showers, has less effect upon the organism: but, if the cloud is so near the earth as to touch it, and we are in that cloud, the electricity may act upon us: and such is a mist or fog. The dampness of the respiratory apparatus enables it to conduct this electricity; so that, by every inspiration, free electricity comes in contact with the blood, and, by a continued action, influences the chemico-vital composition of that fluid. Mists, damp places, are therefore one among the causes of typhus. Diminished *atmospheric pressure* (a low condition of the barometer) diminishes also nervous influence, and becomes, therefore, very favorable to the development of the nervous constituent of typhus. Moisture of the air alone does not develop typhus, but only in connexion with increased atmospheric pressure and electricity.

Applying what has now been said to forms of disease, typhus fever is regarded as a universal Typhoseptosis; the neurasthenia typhica and the lesion of the blood being so far developed as to implicate the whole organism with its power of reaction; whilst, in the local forms, the whole blood is disturbed, (although in a less degree,) but the nervous affection is limited to a single organ. There is a morbid reaction, approaching in its character to inflammation. We also see a disposition to concentration in typhus. From what we have already said, it is unnecessary to allude to the symptoms in typhus fever, which the author quotes as illustrative of his views. The following is his explanation of the affections of the glands of Peyer and Brunner:—The *vis medicatrix* commences (with the development of the lesion of the blood) its endeavour to throw off the albuminous parts; but, if these be in too great quantity, albumen is deposited in the intestinal follicles or between the coats of the intestines, and hence the enlargement of Peyer's and Brunner's glands. If death happen within five days of the attack, and these glands are cut through, the upper layer is found to be mucous membrane perfectly normal, except that, from being stretched by what lies beneath it, it is somewhat thinned. Beneath this membrane is a layer, from one to three lines in thickness, which consists of fibrin, some phosphate of lime, lactate and hydrochlorate of soda, and traces of other salts. A similar process occurs in the glands of the mesentery and mesocolon. From the fifth to the ninth day, ulceration of the mucous membrane takes place, and leaves the whitish matter exposed. This deposition often takes place beneath the vascular membrane; likewise ending in ulceration and discharge. The mucous membrane and ulcerated surface secrete fluid, partly serous, partly membranous, which comes away as diarrhœa: the flocculent part consists of albumen, fibrin, phosphate of lime dissolved in the serous part, which consists of water, salts, and some uncoagulated albumen. As these pathological phenomena are the consequences of reaction of the organism, the nervous power being oppressed, it is clear that they may occur in other diseases where these conditions exist, although not exactly in the same degree as in typhus. Thus are there similar phenomena in Asiatic cholera, in scarlet fever, in phthisis.

Typhus, in its tendency to concentration, shows a great disposition to effect the destruction of an organ. The less developed the Typhosepsis, the nearer is the action to true inflammation. The highest degree of its development is the destruction of the organization of a part,—gangrene. Almost all organs become softened, and some to such a degree that their texture is lost. Such is gelatiniform softening. Typhus presents various appearances, from the greater prevalence of one or more of the phenomena of general Typhoseptosis; so that every epidemic has in it something peculiar to itself. The pathogenic characteristics are, however, never absent, but in various degrees form the groundwork of all its varied phenomena.

According with Buzorini's views of the pathology of typhus are the indications of treatment: 1, to counteract the paralysed state of blood and nerves; 2, to encourage the excretion of deleterious matters from the blood, by the most natural channels; 3, to moderate reaction of the organism; 4, to oppose concentration on individual organs. We shall quote from the author's remarks on remedies those only which relate to ipecacuanha. He says that the radix ipecacuanhæ possesses a specific power of exciting the nervous system without any injurious secondary action, which is contraindicated by the typhoid state; and that it is, consequently, a very valuable remedy in typhus. It acts little, if at all, on the circulation of the blood, whilst it somewhat excites and strengthens its vitality: it consequently increases the activity of the normal excrementitious organs, such as the skin, mucous membranes, liver, salivary glands, and kidneys, and somewhat retards the evacuations by stool. It is proper in all stages of typhus. It moderates the nervous symptoms. cuts them short,—puts an end to mild cases in the beginning, by encouraging excretions,—and acts so favorably on the nervous system that restlessness and delirium often speedily cease after its use, or are very much diminished. Its beneficial action is less evident in worse forms of the disease. Buzorini has employed ipecacuanha in typhus for a period of ten years, with the most favorable effects. The form of its administration is an infusion of from one to two scruples in from five to seven ounces of water, daily. It does not excite vomiting in typhus, as it would do in other conditions; and when this happens it is regarded as a favorable sign.

We have no room for what the author adds on local Typhoseptoses. What we have already given has been partly on account of the application which it contains of electricity to morbid phenomena; a circumstance which, if it be too much regarded by some German physicians, is far too lightly estimated by ourselves; partly because, unless we occasionally afforded our readers a specimen of this kind of foreign medical literature, we could scarcely be regarded as its impartial reviewers. There are so many obvious objections to the theory of Buzorini, that we have merely given it without note or comment: and if, in the application of the theory to typhus fever, there is a want of evidence, much more is this the case with the local forms of what are termed Typhoseptoses: for instance, hospital gangrene; stomacace; diphtheritis; cynanche trachealis; dysenteria; febris puerperalis; hydrocephalus; trismus neonatorum, &c.

